# Rare Prekallikrein Deficiency Identified During Workup of Isolated Prolonged Activated Partial Thromboplastin Time

**DOI:** 10.31486/toj.24.0046

**Published:** 2024

**Authors:** Jennifer S. Woo, Jennifer Tseng, Irene M. Kang, Lefan Zhuang, Ryan Jackson, Olga V. Danilova, Azra Borogovac

**Affiliations:** ^1^ Department of Pathology, City of Hope National Medical Center, Irvine, CA; ^2^ Department of Surgery, City of Hope National Medical Center, Irvine, CA; ^3^ Department of Medical Oncology and Therapeutics Research, City of Hope National Medical Center, Irvine, CA; ^4^ Department of Pathology, City of Hope National Medical Center, Duarte, CA; ^5^ Department of Hematology and Hematopoietic Cell Transplantation, City of Hope National Medical Center, Irvine, CA

**Keywords:** *Blood coagulation*, *hemostasis*, *prekallikrein*

## Abstract

**Background:** Prolongation of the activated partial thromboplastin time (aPTT) may signify an intrinsic factor deficiency or the presence of an inhibitor of coagulation, potentially placing a patient at increased risk for bleeding. However, a contact factor (ie, factor XII, prekallikrein, and high molecular weight kininogen) deficiency, which may also cause a prolonged aPTT, is not associated with clinical bleeding.

**Case Report:** A 71-year-old female had an isolated prolonged aPTT discovered during preoperative laboratory testing. Results of the initial coagulation tests demonstrated no deficiency of factors VIII, IX, or XI and no evidence for lupus anticoagulant or antiphospholipid antibodies. However, a mixing study test was suggestive of factor deficiency. Based on these results, contact factor activity tests were performed to identify any contact factor deficiency. The patient was determined to have a prekallikrein deficiency.

**Conclusion:** Rare causes of isolated prolonged aPTT include contact factor deficiencies such as prekallikrein deficiency. Identification of a contact factor deficiency is clinically useful information, as it allows for a definitive assessment of bleeding risk. This case reports a rare factor deficiency and illustrates a contemporary approach to the workup of an isolated prolonged aPTT.

## INTRODUCTION

Isolated prolonged activated partial thromboplastin time (aPTT) refers to the prolongation of aPTT in the setting of a normal prothrombin time and can be seen in up to 12% of hospitalized patients.^1^ Determining the cause of an isolated prolonged aPTT is necessary to assess a patient's risk for bleeding.

The aPTT test is a clot-based test used to evaluate the intrinsic pathway of coagulation. The coagulation factors that influence aPTT are the intrinsic factors VIII, IX, and XI and the contact factors XII, prekallikrein, and high molecular weight kininogen. Prolongation of the aPTT above the reference range is considered abnormal and may signify an intrinsic or contact factor deficiency or the presence of an inhibitor of coagulation. Inhibitors of coagulation include anticoagulant medications and specific or nonspecific antibodies that neutralize coagulation factors.^2^

Possible causes of an isolated prolonged aPTT include the presence of (1) lupus anticoagulant antibodies, the most common cause of an isolated prolonged aPTT^3^; (2) thrombin-inhibiting anticoagulant medications such as unfractionated heparin or direct thrombin inhibitors^4^; (3) congenital or acquired deficiency of intrinsic factors or contact factors; and (4) intrinsic factor inhibitors.^5^ Deficiencies and inhibitors of intrinsic factors (eg, factors VIII, IX, and XI) are associated with clinical bleeding,^6^ while deficiencies of contact factors are not.^7^

We report the case of a patient with a prekallikrein deficiency, a type of contact factor deficiency, and demonstrate a contemporary approach to the workup of an isolated prolonged aPTT.

## CASE REPORT

A 71-year-old female of European ancestry with breast invasive ductal carcinoma presented for preoperative consultation. Her medical history was significant for gastric carcinoma, and she was status post total gastrectomy 5 years prior. The patient was interested in proceeding with surgery; however, her preoperative laboratory workup demonstrated isolated prolonged aPTT at 121.3 seconds (reference range, 25.4-34.3 seconds). Prothrombin time was normal at 12.5 seconds. Because of concern for increased bleeding risk, the patient was referred to Hematology for further workup. A thorough bleeding history was undertaken; the patient reported no history of abnormal bleeding although she endorsed bruising at the breast biopsy site. Past surgical notes detailed significant postoperative hemorrhage secondary to an arterial bleed following gastrectomy that required surgical intervention and transfusion. The patient's aPTT was noted to be prolonged at the time of gastrectomy, ranging from 47.6 to 75.7 seconds, but the prolonged aPTT was not addressed at the time. The patient had no family history of bleeding and no history of thrombosis or pregnancy loss.

Additional laboratory studies were performed to identify the cause of the patient's isolated prolonged aPTT and to assess whether she could be cleared for surgery (Table). Initial testing included thrombin time, factor activity tests, lupus anticoagulant testing, antiphospholipid antibody testing, and a mixing study test. Thrombin time was normal at 15.6 seconds, ruling out the presence of thrombin-inhibiting medications. Factor activity tests for factors VIII, IX, and XI demonstrated no deficiencies of these factors (158%, 134%, and 168.5%, respectively). Lupus anticoagulant testing, including dilute Russell viper venom time and lupus anticoagulant–sensitive partial thromboplastin time with hexagonal phospholipid neutralization assay, were negative. Antiphospholipid antibody testing, including anticardiolipin (immunoglobulin G [IgG]/immunoglobulin M [IgM]) and beta-2 glycoprotein 1 antibodies (IgG/IgM), were also negative. The mixing study test demonstrated correction of aPTT into the reference range, suggestive of a factor deficiency.

**Table.** Coagulation Workup for Isolated Prolonged Activated Partial Thromboplastin Time

Laboratory Test	Result	Reference Range
Activated partial thromboplastin time, seconds	121.3	25.4-34.3
Prothrombin time, seconds	12.5	12.7-14.5
Thrombin time, seconds	15.6	14-21
Activated partial thromboplastin time mixing study, interpretation	Correction of activated partial thromboplastin time into reference range	N/A
Factor VIII activity, %	158	56-140
Factor IX activity, %	134	50-150
Factor XI activity, %	168.5	50-150
Factor XII activity, %	110.2	50-150
Prekallikrein, %	<15	55-207
High molecular weight kininogen, %	177	65-135
Dilute Russell viper venom time, interpretation	Negative	Negative
Lupus anticoagulant–sensitive partial thromboplastin time with hexagonal phospholipid neutralization, interpretation	Negative	Negative
Anticardiolipin IgG, GPL U/mL	<9	0-14
Anticardiolipin IgM, MPL U/mL	<9	0-12
Beta-2 glycoprotein 1 antibody IgG, GP1 IgG units	<9	0-20
Beta-2 glycoprotein 1 antibody IgM, GP1 IgM units	<9	0-32

IgG, immunoglobulin G; IgM, immunoglobulin M; N/A, not applicable.

Because the mixing study was suggestive of factor deficiency, testing for contact factor activity was performed, as deficiencies of factors VIII, IX, and XI were excluded. Factor XII activity was normal (110.2%). Prekallikrein and high molecular weight kininogen activity tests performed at a specialized reference laboratory demonstrated a deficiency of prekallikrein (<15%, reference range, 55%-207%) and no deficiency of high molecular weight kininogen (177%, reference range, 65%-135%). Given the results of all the tests and the patient's isolated prolonged aPTT in the past, the findings were most consistent with congenital prekallikrein deficiency.

The patient was cleared for surgery and treated per the standard of care. She tolerated the surgery well and did not have any bleeding events either during surgery or postoperatively.

## DISCUSSION

Preoperative coagulation testing of aPTT and prothrombin time are generally discouraged, as contemporary evidence shows coagulation testing is a poor predictor of bleeding and may lead to unnecessary delays in surgery.^8^ Instead, preoperative coagulation studies should be reserved for selected patients, especially those with active bleeding and history of a bleeding disorder.^9,10^ Although test utilization practices are changing in response to current evidence, preoperative coagulation testing may still be ordered, and providers must be familiar with the workup of abnormal coagulation values.

Isolated prolonged aPTT refers to the prolongation of aPTT in the setting of a normal prothrombin time. Determining the cause of isolated prolonged aPTT is necessary to assess bleeding risk and to develop an appropriate therapeutic management plan. Our case highlights a patient with a rare factor deficiency and illustrates a contemporary approach to the workup of isolated prolonged aPTT (Figure).^3,5,11,12^

**Figure.**
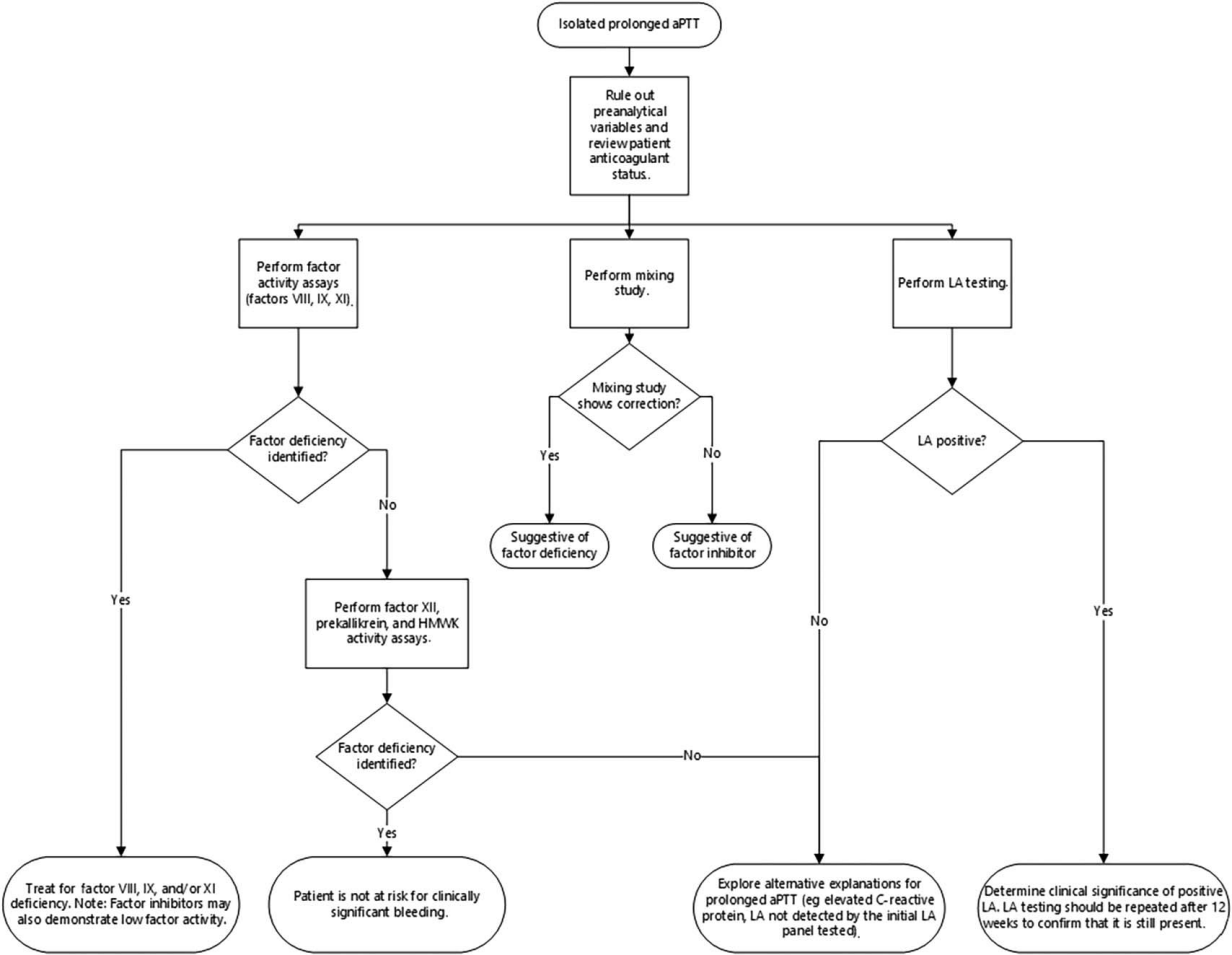
**Diagnostic algorithm for the contemporary workup of an isolated prolonged activated partial thromboplastin time (aPTT). This algorithm depicts factor activity testing, lupus anticoagulant (LA) testing, and the mixing study test performed concurrently.**^3^^,^^5^^,^^11^^,^^12^ HMWK, high molecular weight kininogen.

The workup begins with ruling out preanalytical causes of prolonged aPTT. Preanalytical causes include heparin contamination through an intravenous line, collection of serum or a clotted sample, and/or altered plasma to citrate ratio due to hemoconcentration (hematocrit >55%) or incorrectly filled collection tubes.^5^ Information regarding the patient's anticoagulant status is critically important, and whenever possible, specimens should be collected in patients not receiving any anticoagulant treatment. The thrombin time test can be elevated in the presence of thrombin-inhibiting anticoagulant medications. Therefore, the thrombin time test can be performed as a screening test to rule out interference with thrombin-inhibiting anticoagulant drugs.^5^

In the medical curriculum, the mixing study has traditionally been taught as the initial test to differentiate between factor deficiency and the presence of an inhibitor. However, in clinical practice, mixing study tests are often conducted concurrently with other coagulation tests or omitted entirely.^13^ The contemporary approach to investigating an isolated prolonged aPTT prioritizes evaluating for factor deficiency, a clinically relevant diagnosis, and assessing for the presence of lupus anticoagulant antibodies, the most common cause of isolated prolonged aPTT.^3^ While the mixing study test is less emphasized, it may still offer valuable insights for guiding a hemostatic workup. Consequently, factor activity testing, lupus anticoagulant testing, and mixing study tests are performed concurrently in the contemporary diagnostic algorithm, rather than following a stepwise approach starting with the mixing study.

Factor activity tests are performed in the workup of an isolated prolonged aPTT to determine whether a factor deficiency or inhibitor is present. Factor activity tests for factors VIII, IX, and XI should be performed because deficiencies of these factors are associated with clinical bleeding. Factor activity tests can also suggest the presence of inhibitors, as inhibitors can cause factor activity to appear low or demonstrate an inhibitor pattern during testing (ie, factor activity appears to increase with each dilution).^5^ If an inhibitor pattern is identified or is suspected for a specific factor, factor inhibitor testing can be performed to confirm the presence of the specific factor being investigated. A noteworthy point is that patients with von Willebrand disease may also present with an isolated prolonged aPTT because of the reduced factor VIII causing the aPTT prolongation.^14^ Factor VIII levels may be reduced in patients with von Willebrand disease because von Willebrand factor protects factor VIII from proteolysis.^15^ Therefore, workup for von Willebrand disease may be appropriate for patients with both isolated prolonged aPTT and reduced factor VIII; however, aPTT is not a reliable test in screening for a diagnosis of von Willebrand disease.^16^

Lupus anticoagulants are a heterogeneous group of antibodies directed against phospholipid-binding proteins, thereby prolonging phospholipid-dependent coagulation assays. Clinically, however, lupus anticoagulants are associated with increased risk for thrombosis and not with anticoagulation in vivo as the name suggests. Because lupus anticoagulants are the most common cause of an isolated prolonged aPTT, lupus anticoagulant testing should be performed to investigate prolonged aPTT. Examples of lupus anticoagulant tests include the dilute Russell viper venom time and aPTT-based tests utilizing a low concentration of phospholipid.^11^ In the workup of antiphospholipid antibody syndrome, lupus anticoagulant testing should be performed together with tests for anticardiolipin and beta-2 glycoprotein 1 antibodies.^11^

Mixing studies are performed by mixing an equal volume of citrated patient plasma with normal pooled plasma. The 1:1 mix is then tested for aPTT both immediately after mixing and after a 1- to 2-hour incubation at 37 °C to assess for time- and temperature-dependent inhibitors. Because only 30% of factor activity is sufficient to yield a normal aPTT result, the 1:1 mixture should allow for the aPTT of the mix to correct into the normal reference range if a sample is factor deficient.^5^ Conversely, an aPTT mixing study that does not correct suggests the presence of an inhibitor. Lupus anticoagulants act as inhibitors of coagulation and therefore demonstrate a mixing study that does not correct.^5^ In many instances, a mixing study is bypassed altogether, as mixing studies are not standardized and can be difficult to interpret,^13^ such as in instances of near-complete correction in the setting of multiple factor deficiencies or correction in the setting of a weak lupus anticoagulant that is diluted out with the addition of normal plasma. For some providers, the mixing study serves as a secondary screening assay, while specific factor activities, factor inhibitor studies, and lupus anticoagulant testing allow for definitive diagnosis.^13^

If deficiencies and inhibitors of factors VIII, IX, and XI are excluded, and a mixing study demonstrates correction, measurement of contact factors can be performed to evaluate for contact factor deficiency. Benzon et al reported that factor XII deficiency was the most frequent factor deficiency identified in adult patients with isolated prolonged aPTT, while deficiencies of prekallikrein and high molecular weight kininogen are uncommon.^17^ Contact factor tests are typically performed at specialized reference laboratories. In general, providers must weigh the benefits of additional testing against the potential delay of treatment. Some physicians may argue that the measurement of contact factors does not add significant value once lupus anticoagulants and deficiencies of factors VIII, IX, and XI are excluded.

If factor deficiencies/inhibitors and lupus anticoagulants are excluded, alternate causes for a prolonged aPTT can be explored, such as elevated C-reactive protein displaying phospholipid-dependent interference with aPTT^18^ or lupus anticoagulants not detected by the lupus anticoagulant tests because other variables influenced the testing. For instance, acute phase reactants, such as elevated factor VIII, shorten the aPTT and may mask lupus anticoagulants during lupus anticoagulant testing.^19^

Plasma prekallikrein, also known as Fletcher factor, is a contact factor that participates in the initiation of the intrinsic coagulation pathway. In circulation, prekallikrein is mainly complexed with high molecular weight kininogen.^20-22^ As a zymogen, prekallikrein is activated to the serine protease kallikrein by activated factor XII (XIIa), with high molecular weight kininogen as a cofactor. Kallikrein in turn initiates surface-mediated activation of coagulation and participates in fibrinolysis and kinin generation.^22^

Deficiencies of prekallikrein may result in prolongation of the aPTT. Congenital prekallikrein deficiency is a rare autosomal recessive condition associated with mutations of the KLKB1 gene.^21^ In a 2020 study, Barco et al analyzed 111 individuals with prekallikrein deficiency, the largest cohort to date.^23^ The authors estimated the prevalence of severe prekallikrein deficiency to be 1/155,668 overall and 1/4,725 for individuals of African ancestry. The study also demonstrated a low prevalence of bleeding events, consistent with the understanding that clinical bleeding is not associated with prekallikrein deficiency.^23^ In general, genetic testing for KLKB1 mutations is not required in routine practice.

## CONCLUSION

We report the case of a patient with a rare prekallikrein deficiency that was identified during workup of an isolated prolonged aPTT. Evaluation for contact factor deficiencies can be considered if the mixing study is suggestive of a factor deficiency but intrinsic factor activities are normal. The contemporary workup of an isolated prolonged aPTT emphasizes workup for factor deficiencies and the presence of lupus anticoagulants. The mixing study test is less emphasized, but it may still provide useful information to direct a hemostatic workup.

## Notes

*This article meets the Accreditation Council for Graduate Medical Education and the American Board of Medical Specialties Maintenance of Certification competencies for Patient Care and Medical Knowledge.*
